# A data-driven indicator for assessing the evolving impact of the EU Common Agricultural Policy on soil erosion mitigation

**DOI:** 10.1016/j.dib.2025.112390

**Published:** 2025-12-13

**Authors:** Pasquale Borrelli, Panos Panagos

**Affiliations:** a Department of Science, Roma Tre University, Rome, 00154, Italy; b Environmental Geosciences, University of Basel, Basel, 4056, Switzerland; c European Commission, Joint Research Centre (JRC), Ispra, Italy

**Keywords:** Soil conservation, Tillage, Crop residues, Cover crop, RUSLE-type modeling, Policy support

## Abstract

The data presented here correspond to the updated LAND Use and Management (LANDUM) model, a core component of the European Commission’s RUSLE-based soil erosion risk assessment framework. LANDUM functions as a data-driven indicator for evaluating the effects of regional land use and agricultural management practices, including measures promoted under the Common Agricultural Policy, on soil erosion intensity at the NUTS2 level within the European Union. The approach relies on spatially explicit estimates of the cover-management (C) factor, a key component of the (R)USLE family of models. In the latest revision presented here, data from the 2023 EU Farm Structure Survey were incorporated to capture the extent of conservation practices such as reduced tillage, the use of cover crops, and the retention of crop residues. These data were processed to assess changes in the C-factor across Europe between 2016 and 2023. Collectively, the four versions of the LANDUM data-driven indicator here reported enable tracking of the European Union’s (EU) Common Agricultural Policy (CAP) effects, from the no-management, pre-GAEC (Good Agricultural and Environmental Condition, introduced with the 2003 CAP reform) baseline in 2000 through the years 2010, 2016, and 2023. The insights gained from the data illustrate both overall and regional trends in how soil conservation measures promoted under the EU CAP contribute primarily to the mitigation of water-driven soil erosion, as well as to the reduction of wind erosion and other related soil degradation processes. These data can support further research in soil erosion and related fields and are available for reuse, reprocessing, or integration to enhance modelling applications that incorporate soil cover and management practices as input variables.

Specifications Table

**Table:** 

Subject	Earth & Environmental Sciences
Specific subject area	Impact of agricultural and soil management practices on soil erosion mitigation
Type of data	Processed (Figures (maps) GeoTIFF format) [1]
Data collection	Data from the Farm Structure Survey (FSS) were obtained from Eurostat [2,3] under *Agriculture → Farm Structure → Management and Practices*. We extracted indicators related to soil tillage (conventional, conservation, zero tillage), crop-management practices (winter cover or intermediate crops, plant-residue management), and arable-land crop statistics for the 27 EU Member States. The datasets were downloaded in Excel format and aggregated at the NUTS2 administrative level for the years 2010, 2016, and 2023 (retrieved in September 2025). To delineate arable land across the EU, we used the 100-m CORINE Land Cover datasets for 2006, 2012, and 2018, downloaded as GeoTIFFs from the Copernicus Land Monitoring Service [4]. These grid data were used to extract arable-land classes and to spatially align land-cover information with the administrative units used in the FSS. RUSLE C-factor parameters for the relevant crop and soil-management categories were derived from the LANDUM inventory, available through the European Soil Data Centre (ESDAC) [5]. The LANDUM values were linked to the corresponding NUTS2 regions to support consistent parametrization across the study area.
Data source location	The Farm Structure Survey data were obtained from the Eurostat Data Explorer (https://ec.europa.eu/eurostat/web/main/data/database), under the category *Agriculture → Farm Structure → Management and Practices*. CORINE Land Cover 100-m raster datasets were downloaded from the Copernicus Land Monitoring Service (https://land.copernicus.eu/en/products/corine-land-cover). RUSLE C-factor information was sourced from the LANDUM dataset available through the European Soil Data Centre (ESDAC) at https://esdac.jrc.ec.europa.eu/content/cover-management-factor-c-factor-eu All datasets are publicly accessible and provided in standard open-data formats (Excel and GeoTIFF).
Data accessibility	Repository name: ZENODO Data identification number: 10.5281/zenodo.17668418 Direct URL to data: https://zenodo.org/records/17668418
Related research article	P. Borrelli, P. Panagos, An indicator to reflect the mitigating effect of Common Agricultural Policy on soil erosion, Land use policy 92 (2020), 104,467, 1–8. https://doi.org/10.1016/j.landusepol.2020.104467

## 1 Value of the Data

• The LAND Use and Management (LANDUM) model is a core component of the EU agri-environmental soil-erosion indicator, used to monitor the mitigating effects of the Common Agricultural Policy (CAP). This dataset provides a policy-relevant and spatially explicit resource for assessing soil-erosion mitigation potential across the 27 EU Member States from 1990 to 2023. By combining regional land use, crop statistics, and actual management practices (reduced or zero tillage, cover cropping, residue retention) with RUSLE C-factor parameters from LANDUM, it enables quantification of how management decisions influence erosion risk at NUTS2 level. The dataset also supports the analysis of long-term trends in management-driven mitigation and more robust forecasting of future erosion risk under evolving agricultural practices.
• Researchers can use the dataset to analyze temporal trajectories of erosion-mitigation potential and track how conservation practices have changed under the CAP. Its spatial resolution allows comparison across regions to identify hotspots of mitigation potential or persistent erosion risk, informing region-specific conservation strategies. The dataset can also be integrated with soil, climate, or ecosystem-services data to assess broader implications or proxies for soil health, water regulation, nutrient retention, and long-term land productivity.
• For policymakers and regional land managers, the dataset offers actionable insights into the effectiveness of agri-environmental measures, supports the targeted design of soil-conservation policies, and helps prioritize regions where management interventions would yield the greatest benefit. In doing so, it strengthens evidence-based decision-making for sustainable land management and soil preservation across the EU.

## 2 Background

Soil erosion remains a significant threat to agricultural productivity and ecosystem services across the European Union [6]. Policy interventions, such as the Good Agricultural and Environmental Conditions (GAEC) standards introduced under the EU Common Agricultural Policy (CAP) in 2003, have introduced conservation-oriented practices, including terraces, grass margins, buffer strips, and reduced tillage, to mitigate soil loss. A European Commission analysis reported that between 2000 and 2010, CAP-driven measures contributed to reducing water-driven soil erosion [7]. Despite these improvements, soil formation rates in many regions still fail to keep pace with erosion, highlighting the need for continuous monitoring to ensure long-term soil health. To assess soil degradation and policy effectiveness, the LAND Use and Management (LANDUM) model was integrated into the European Commission’s RUSLE-based pan-European soil erosion platform [8,9]. This high-resolution (100 m) model provides regionally explicit estimates of the cover-management (C) factor, a unitless indicator of land use and management effects on soil erosion across NUTS2 regions. Changes in the C factor over time indicate shifts in management and policy impacts. A dataset is presented documenting the effects of management changes, including reduced tillage, cover crops, and crop residue retention, enabling trend assessment for 1990, 2010, 2016, and 2023. This publicly available dataset aims to support decision-makers by extending current knowledge and, from a scientific perspective, to contribute to the development and refinement of modelling tools for evaluating soil degradation caused by erosion.

## 3 Data Description

**Fig. 1:**
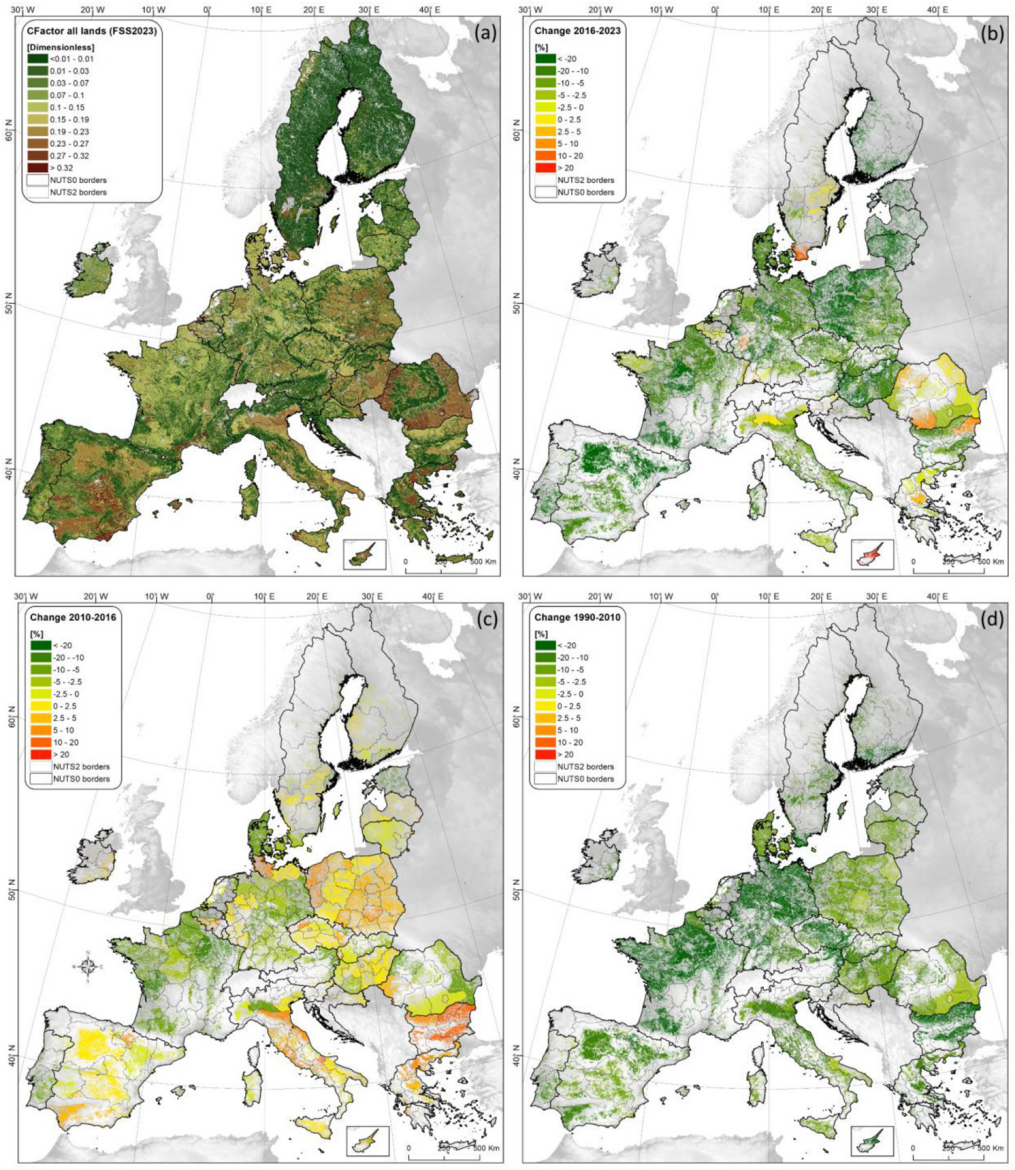
Spatial distribution of the cover-management factor (C-factor; dimensionless) in erodible lands of the European Union for 2023, derived from LANDUM data (a), and sub-national (NUTS-2 level) changes in the average C-factor for arable land during the periods 2016–2023 (b), 2010–2016 (c), and 1990–2010 (d).

**Fig. 2:**
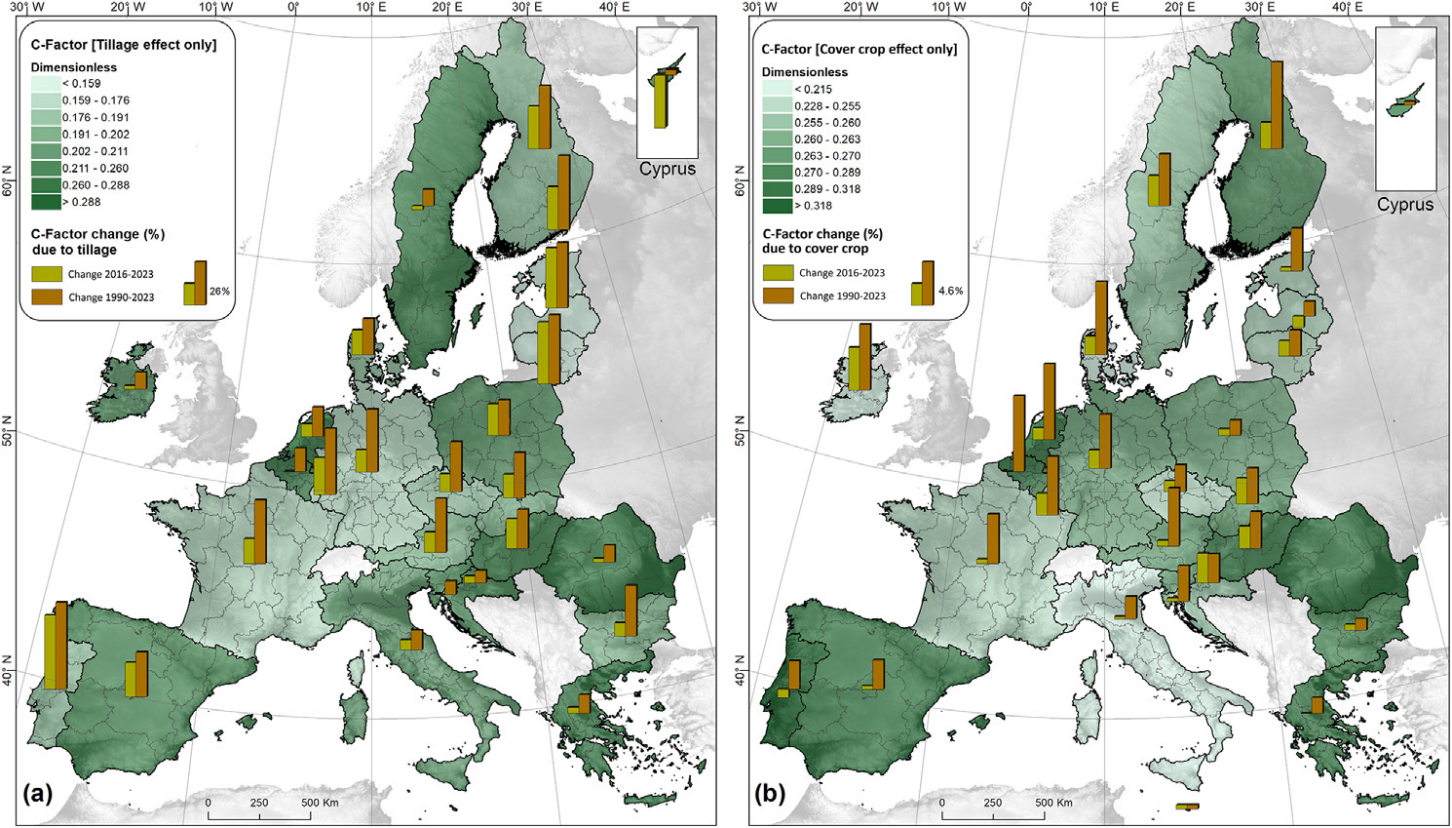
Spatial distribution of the average national cover-management factor (C-factor; dimensionless) across the European Union, considering only the reduction effects of (a) tillage management and (b) winter cover crops. The bars represent the percentage changes between the periods 1990–2010, 2010–2016, and 2016–2023.

Pan-European maps estimating the cover-management (C) factor for soil erosion modelling are provided for pre-GAEC (2000, under the assumption that no soil conservation measures were in place) and post-GAEC (2010, 2016, and 2023) periods. Fig. 1a shows the results for 2023, divided into ten classes following the European Soil Bureau classification, while Fig. 1b–c illustrate percentage changes between 2016 and 2023, 2010–2016, and 1990–2010, reflecting the evolution of crop management practices across NUTS3 administrative units (as reported in the Eurostat Farm Structure Survey). Table 1 provides an overview of temporal changes in conservation measures promoted under the EU CAP, while Fig. 2 illustrates changes in conservation tillage (reduced tillage plus no-till) and winter cover (cover crops or intermediate crops).

**Table 1:** Descriptive statistics for crop management types based on data from the EU Farm Structure Survey.

Crop management type	Arable land by type			Change	
[EU27]	2023	2016	2010	2023–2016	2016–2010
	[million hectares]			[ %]	
Arable land	96.92	93.57	94.30	3.6	−0.8
Arable land excluding soil cover	8.37	7.52	14.71	11.3	−48.9
Normal winter crop	53.15	43.53	41.39	22.1	5.2
Cover crop or intermediate crop	10.25	7.15	5.44	43.4	31.3
Plant residues	9.25	6.67	8.28	38.6	−19.4
Bare soil	15.82	21.38	24.47	−26.0	−12.7
Arable land excluding tillage	11.89	9.44	12.67	25.9	−25.5
Conventional tillage	52.15	62.49	61.01	−16.5	2.4
Conservational tillage	25.69	18.22	17.43	41.0	4.6

A table is provided (Excel file, .xlsx) and spatial files, including shapefiles (.shp) and gridded raster files (.tif), for both absolute values and period-to-period changes. The gridded raster provides continuous data at a 100 m resolution, spatially aligned with CORINE Land Cover data, and referenced to the INSPIRE-compliant European Terrestrial Reference System 1989 (ETRS89) projected coordinate system.

Relative to a no-conservation (pre-GAEC) baseline (2000), the average C-factor for EU-27 (excluding the United Kingdom) arable land decreases by approximately 28.6 % in 2023, a substantially larger reduction than the 16.3 % and 15.8 % observed in 2016 and 2010, respectively. This indicates that the soil conservation practices implemented between 2016 (C-factor = 0.2355) and 2023 (C-factor = 0.2011) led to a 14.6 % decrease in this soil-erosion indicator, which is considerably greater than the minimal change recorded between 2010 (C-factor = 0.2369) and 2016 (–0.6 %).

The conservation practices driving the increased soil-erosion mitigation potential outlined above have grown substantially across Europe. In 2023, conservation tillage remained the dominant measure, applied on 26.5 % of arable land (about 96.9 million ha), representing a ∼41 % increase since 2010 (from 18.2 to 25.7 million ha). No-till adoption also rose markedly (+111.1 %), reaching 7.4 % of arable land in 2023 compared with 3.6 % in 2016. Cover-crop use expanded from 7.6 % to 10.6 % between 2016 and 2023, a 2.9-percentage-point rise slightly higher than the 2.4-point increase observed from 2010 to 2016. The use of plant residues increased by 38.6 %, covering 9.5 % of arable land in 2023 compared with 7.1 % in 2010. Over the same period, bare-soil conditions declined by 15.7 percentage points, falling from 21.4 million ha in 2016 to 15.8 million ha in 2023. Consequently, the area without any soil cover dropped to 8.37 million ha (8.6 % of arable land).

## 4 Experimental Design, Materials and Methods

### 4.1 Data collection

Raw data on ‘*Soil cover by arable land area, farm type, and crop rotation*’ [2] and ‘*Soil management practices by arable land area, farm type, and crop rotation*’ [3], derived from the EU Farm Structure Survey, were acquired in Excel format from the Eurostat database (https://ec.europa.eu/). Territorial units for statistics (NUTS) data were also obtained from the Eurostat repository [10] under the following conditions: year = 2024, format = shapefile, geometry = polygons, scale = 1:1 M, coordinate reference system = EPSG:3035.

Base values of the cover-management (C) factor for arable land and semi-natural vegetation were obtained from available sources and supplemented with MERIS satellite data on the percentage of soil covered by vegetation. These data, were accessed from the European Commission’s Soil Data Centre (ESDAC) (https://esdac.jrc.ec.europa.eu/).

### 4.2 Cover-management (C) factor estimates

**Fig. 3:**
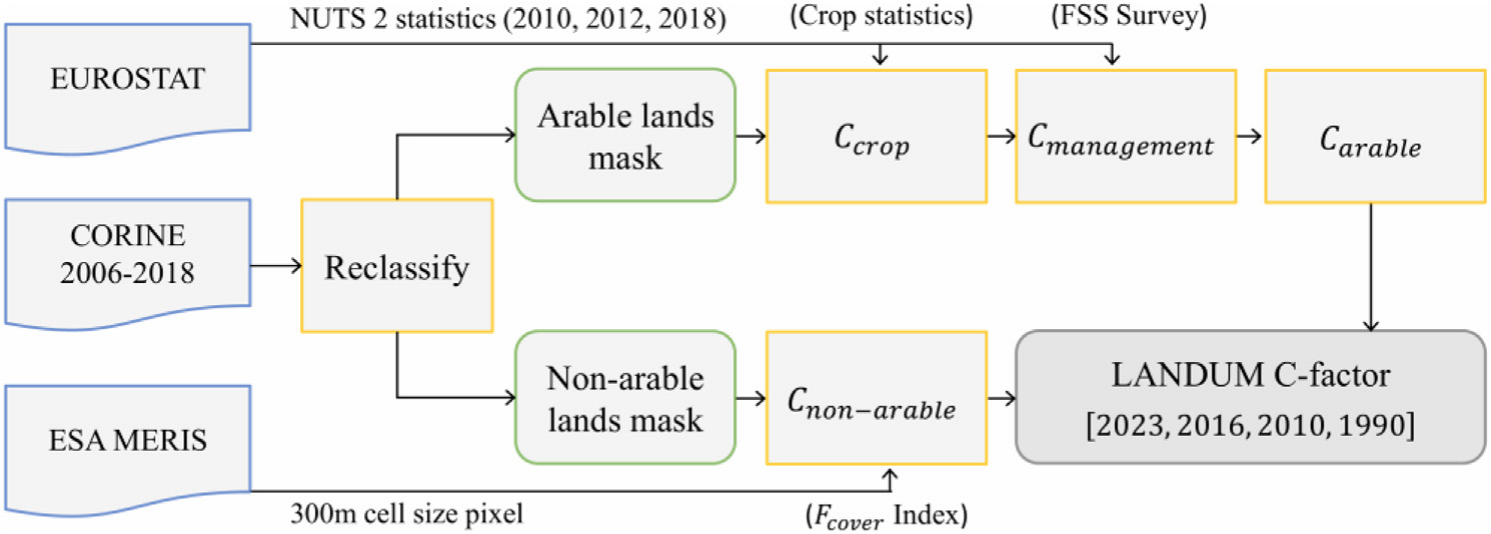
Conceptual diagram illustrating the key processing stages used to evaluate the C-factor within LANDUM. The *arable land* category corresponds to CORINE Land Cover classes 2.1.x (arable land), while *non-arable land* encompasses classes 2.2.x (permanent crops), 2.3.1 (pastures), 2.4.x (heterogeneous agricultural areas), 3.1.x (forests), 3.2.x (scrub and/or herbaceous vegetation), and 3.3.x (open areas with sparse or no vegetation).

The LANDUM model estimates annual C-factor values for arable and non-arable lands using two approaches (Fig. 3). Non-erosive areas such as bare rocks, wetlands, glaciers, water bodies, and artificial surfaces, which account for ∼10 % of EU land, are excluded. Arable and non-arable lands are defined using the pan-European CORINE Land Cover 2006 database at 100 m resolution. CORINE, has 44 classes and a minimum mapping unit of 25 ha. Land cover changes between 2006, 2012, and 2018 were integrated using the official CORINE Land Cover Change dataset to maintain comparability with previous LANDUM versions, as advised in CORINE metadata.

**C-factor for arable lands** was estimated for land under temporary crops using the CORINE Land Cover map (2006, 2012, 2018) using the categories: non-irrigated arable land (code = 2.1.1); permanently irrigated (code = 2.1.2), and rice fields (code = 2.1.3). Crop distribution across European arable lands was derived from Eurostat statistics at the NUTS2 level. A five-year dataset (2008–2012) covering sixteen crops plus fallow land was used to describe crop rotations for the 2010 and 2016 scenarios. The same crop rotations were assumed for the 2023 scenario. However, to capture recent changes in crop dynamics, such as the increase in corn for biofuel production, LANDUM was rerun using crop rotation data for the 2019–2023 period, allowing these shifts to be incorporated into the assessment. C-factor values for individual crops were based on experimental data [8] and combined within each NUTS2 as a weighted average:

(1) $$C_{crop}=\sum_{n=1}^{17}C_{crop\;n}\;\cdot \left[\%\right]NUTS2_{crop\;n}\;$$

Conservation practices (improved tillage, winter cover crops, and crop residues) were included as management factors ($C_{\text{management}}$) following [11]. The overall C-factor for arable land per NUTS2 was computed as:

(2) $$C_{arable}=\;C_{crop}\cdot \;C_{management}$$

where $C_{management}$ is computed as:

(3) $$C_{management}=\;C_{tillage}\cdot C_{cover}\cdot C_{residues}$$

Weights for $C_{\text{tillage}\;}$(1 for conventional, 0.35 for conservation/ridge, 0.25 for no-till), $C_{\text{cover}\;}$(0.80), and $C_{\text{residues}\;}$(0.88) were based on the fraction of land under each practice according to the 2010, 2016 and, 2023 EU Farm Structure Survey statistics.

**The C-factor for non-arable lands** was assigned to CORINE 2006 classes with natural vegetation (permanent crops 2.2.x, pastures 2.3.1, heterogeneous agricultural areas 2.4.x, forests 3.1.x, scrub/herbaceous 3.2.x, open spaces 3.3.x), taking into account land cover changes observed in 2006, 2012, and 2018. Pixel-level values were derived using a semi-qualitative approach, combining literature-based ranges ($C_{landuse}$) with sub-pixel estimates of vegetation cover from the MERIS $F_{cover}$ product, which ranges from 0 (no cover) to 1 (dense canopy). The C-factor was calculated as:

(4) $$C_{non-arable}=Min\left(C_{landuse}\right)+Range\left(C_{landuse}\right)\cdot \left(1-\;F_{cover}\right)$$

$C_{non-arable}$ reaches its maximum when $F_{cover}$ = 0 and its minimum when $F_{cover}$ = 1. To avoid seasonal biases, non-arable lands unchanged between CORINE 2006 and 2012 retain the same C-factor per pixel [8], and new C-factor values are only computed for arable lands reflecting land cover changes between 2006 and 2012.

## Limitations

The LANDUM model represents a major advancement over previous EU-scale C-factor assessments by integrating crop composition, conservation management practices, and remotely sensed vegetation status into the estimates. This approach allows a more realistic representation of spatial variability, for example, the higher vegetation density in pastures in Ireland compared to Cyprus, or herbaceous protection in vineyards of northern Europe compared to bare vineyards in Spain. However, some uncertainties remain. C-factor values for arable lands are aggregated at the NUTS2 or NUTS3 level, meaning the precise location of specific crop types is unknown. While variations are likely minor, estimates could differ from scenarios where exact crop locations are available. Although this limitation could be addressed using recent high-resolution Copernicus data [12], such as Crop Type 2017 to 2023 at 10 m resolution from Sentinel imagery [13], such datasets are unavailable for 1990, 2010, and 2016. For statistical consistency, LANDUM therefore relies on aggregated NUTS-level crop statistics, which constrains spatial precision but ensures comparability across the temporal series. The same limitation that affects crop type data also applies to conservation practices. Information on reduced tillage, no-till, cover crops, and crop residue retention is only available at the NUTS2 level. This prevents a precise assessment of their effects at specific locations, as the impact is averaged across the entire NUTS2 region. In principle, this limitation could be addressed because the 2010, 2016, and 2023 EU Farm Structure Survey data are delocalized and could be allocated at a sub-NUTS2 level. However, for privacy reasons, sub-NUTS2 geolocated data on farm management are currently not made available for scientific purposes, which restricts the spatial resolution at which conservation practice effects can be assessed.

## Ethics Statement

The authors read and followed the ethical requirements for publication in Data in Brief and confirmed that the current work did not involve human subjects, animal experiments, or any data collected from social media platforms.

## Credit Author Statement

Pasquale Borrelli: Data collecting, Data curation, Methodology, Writing Original Draft, Funding Acquisition. Panos Panagos: Methodology, Reviewing and Editing.

## Data Availability

ZENODORaster (GeoTIFF) (Original data).
